# Targeting human leukocyte antigen G with chimeric antigen receptors of natural killer cells convert immunosuppression to ablate solid tumors

**DOI:** 10.1136/jitc-2021-003050

**Published:** 2021-10-18

**Authors:** Chia-Ing Jan, Shi-Wei Huang, Peter Canoll, Jeffrey N Bruce, Yu-Chuan Lin, Chih-Ming Pan, Hsin-Man Lu, Shao-Chih Chiu, Der-Yang Cho

**Affiliations:** 1 Department of Pathology, China Medical University Hospital, Taichung, Taiwan; 2 Department of Medicine, China Medical University, Taichung, Taiwan; 3 Translational Cell Therapy Center, China Medical University Hospital, Taichung, Taiwan; 4 Institute of New Drug Development, China Medical University, Taichung, Taiwan; 5 Department of Pathology and Cell Biology, Columbia University, New York, New York, USA; 6 Department of Neurosurgery, Columbia University, New York, New York, USA; 7 Drug Development Center, China Medical University, Taichung, Taiwan; 8 Department of Psychology, Asia University, Taichung, Taiwan; 9 Graduate Institute of Biomedical Sciences, China Medical University, Taichung, Taiwan; 10 Department of Neurosurgery, China Medical University Hospital, Taichung, Taiwan

**Keywords:** immunotherapy, killer cells, natural, drug therapy, combination

## Abstract

**Background:**

Immunotherapy against solid tumors has long been hampered by the development of immunosuppressive tumor microenvironment, and the lack of a specific tumor-associated antigen that could be targeted in different kinds of solid tumors. Human leukocyte antigen G (HLA-G) is an immune checkpoint protein (ICP) that is neoexpressed in most tumor cells as a way to evade immune attack and has been recently demonstrated as a useful target for chimeric antigen receptor (CAR)-T therapy of leukemia by in vitro studies. Here, we design and test for targeting HLA-G in solid tumors using a CAR strategy.

**Methods:**

We developed a novel CAR strategy using natural killer (NK) cell as effector cells, featuring enhanced cytolytic effect via DAP12-based intracellular signal amplification. A single-chain variable fragment (scFv) against HLA-G is designed as the targeting moiety, and the construct is tested both in vitro and in vivo on four different solid tumor models. We also evaluated the synergy of this anti-HLA-G CAR-NK strategy with low-dose chemotherapy as combination therapy.

**Results:**

HLA-G CAR-transduced NK cells present effective cytolysis of breast, brain, pancreatic, and ovarian cancer cells in vitro, as well as reduced xenograft tumor growth with extended median survival in orthotopic mouse models. In tumor coculture assays, the anti-HLA-G scFv moiety promotes Syk/Zap70 activation of NK cells, suggesting reversal of the HLA-G-mediated immunosuppression and hence restoration of native NK cytolytic functions. Tumor expression of HLA-G can be further induced using low-dose chemotherapy, which when combined with anti-HLA-G CAR-NK results in extensive tumor ablation both in vitro and in vivo. This upregulation of tumor HLA-G involves inhibition of DNMT1 and demethylation of transporter associated with antigen processing 1 promoter.

**Conclusions:**

Our novel CAR-NK strategy exploits the dual nature of HLA-G as both a tumor-associated neoantigen and an ICP to counteract tumor spread. Further ablation of tumors can be boosted when combined with administration of chemotherapeutic agents in clinical use. The readiness of this novel strategy envisions a wide applicability in treating solid tumors.

## Introduction

Chimeric antigen receptors (CARs) for adoptive cell therapy have shown remarkable clinical responses in adult and pediatric patients with hematological malignancies; these successes led to the US Food and Drug Administration approving the use of four new CD19-specific drugs.^1–4^ However, the success of CAR-T therapy in treating B-cell malignancies has yet to translate to solid tumors.^5^ An obstacle to CAR-T activity against solid tumors is the immunosuppressive tumor microenvironment (TME), which leads to inadequate immune cell trafficking and poor/weak cytotoxic immune responses; it also lacks highly expressed uniform tumor antigen among the heterogeneous tumor cells, which results in immune escape variants.^6^ Targeting the immunosuppressive TME is essential if we are to improve CAR-T therapy for solid tumors.^7^ A common strategy used by tumor cells to upregulate programmed death-ligand 1 (PD-L1) and evade immunosurveillance is to create an immunosuppressive TME.^8^ Thus, using CAR against immune checkpoint protein (ICP) molecules is a potential strategy to overcome the hostile TME by converting immune-inhibitory signals to activation signals. Recent studies report that targeting PD-L1 expressed by pancreatic and non-small lung cell tumors have achieved positive results both in vitro and in experimental models^9–12^; however, a recent clinical case report has shown that PD-L1 CAR-T causes adverse events.^13^ Therefore, the most ideal ICP for use as a CAR target should also be a common tumor-associated antigen (TAA) which is widely expressed among heterogeneous tumor cells, which not only may give an opportunity to overcome TME but also may cause fewer severe off-tumor effects in healthy tissues.

Human leukocyte antigen G (HLA-G) is a neoexpressed TAA on 40%–100% of solid tumors.^14 15^ A recent study reports that HLA-G is a potent ICP for CAR-T targeting of hematological cancers.^16^ HLA-G is a non-classical HLA class Ib molecule which exerts potent immunosuppressive activity under different pathophysiological conditions.^17^ Only a limited subset of immune privileged normal tissues (eg, the cornea) and adult tissues, such as erythroid precursors and pancreatic islets, express HLA-G.^18 19^ HLA-G inhibits both innate and adaptive immunity by binding directly to lymphocyte immunoglobulin-like receptor B1 (LILRB1) and killer cell immunoglobulin-like receptor 2DL4 (KIR2DL4) on natural killer (NK) cells and mediated inhibitory signals via their immunoreceptor tyrosine-based inhibition motif, thereby suppressing the cytotoxic functions of NK cells.^20–22^ CAR-NK has several advantages over CAR-T cells, such as CAR-independent tumor killing capacity and safety of allogeneic transplantation potential.^23 24^ However, recent studies have shown that upregulation of HLA-G renders tumor cells resistant to NK cell-based immunotherapies.^25–27^ Thus, HLA-G may play a vital role to impair the chances of successful treatment with CAR-NK therapies.

By contrast, some conventional cytotoxic cancer chemotherapeutic agents and tyrosine kinase inhibitors, such as doxorubicin (Dox) and sunitinib, eradicate tumor cells and increase immunoactivity in the TME simultaneously by activating immunogenic cell death or promoting type-I cytokine responses.^28 29^ While most clinical data show limited efficacy of CAR monotherapy targeting carbonic anhydrase IX (CAIX) or epidermal growth factor receptor (EGFR) for treating solid tumors,^30 31^ combined therapies appear to increase the antitumor potency of CAR-T cell therapy by overcoming the immunosuppressive TME or by enhancing T-cell responses.^32 33^

HLA-G CAR shows antitumor activity both in vitro and in vivo in an HLA-G-expressing hematopoietic tumor model based on K562 and JEG-3 cells.^16^ However, it is not clear whether the antitumor efficacy of HLA-G-redirected CAR is effective in solid tumor models. This study aimed to use a combined approach involving application of low-dose chemotherapy to increase membranous expression of HLA-G by solid tumor cells and then targeting it with HLA-G CAR-NK cells. The results show that pretreatment with low-dose chemotherapy to induce overexpression of HLA-G increases the antitumor efficacy of HLA-G CAR-NK cells both in vitro and in vivo. Furthermore, we also investigated how CAR converted inhibitory HLA-G to activating signal and explained the mechanism of chemotherapy induced cell surface HLA-G on tumor cells.

## Materials and methods

### Reagents and antibodies

BX795 was acquired from Selleckchem. The chemotherapeutic drugs Dox, temozolomide (TMZ), gemcitabine (Gem), carboplatin (CBP), 5-azacytidine (5-aza), and the chemicals protamine sulfate, puromycin dihydrochloride, brefeldin A (BFA), and protein L were purchased from Sigma-Aldrich. AP20187 was purchased from Clontech Laboratories. Antihuman pan-cadherin (28E12), E-cadherin (24E10), DNA methyltransferase 1 (DNMT1) (D63A6), phospho-SHP-1 (Tyr564) (D11G5) and phospho-Zap-70 (Tyr319)/Syk (Tyr352) antibodies were obtained from Cell Signaling Technology. Antibodies specific for human HLA-G (4H84), TAP1 (B-8), signal peptide peptidase (SPP) (ab247061) and β-actin (C-2) were from Santa Cruz Biotechnology. Fluorophore-conjugated or non-conjugated antibodies specific for HLA-G (MEM-G/9), CD107a (1D4B), Fas ligand (NOK-1), and TRAIL (N2B2) were obtained from Abcam. CD45 (2D1), CD56 (MEM-188, fluorescein isothiocyanate (FITC)conjugate), CD16 (CB16), CD3 (OKT3), CD19 (HIB19), CD11b (ICRF44), CD66b (G10F5), CD94 (DX22), NKG2D (1D11), Nkp44 (44.189), Nkp46 (9E2), and Nkp30 (AF29-4D12) were obtained from Thermo Fisher Scientific. The NKG2A (S19004C) and CD56 (MEM-188, PE-conjugated) antibodies was purchased form Biolegend.

### Cell lines

All cell culture media were supplemented with 10% fetal bovine serum (FBS) (Thermo Fisher Scientific). MDA-MB-231 (American Type Culture Collection (ATCC)) and Luc-expressing MDA-MB-231 cell line (My BioSource), AsPC-1 (ATCC), and BEAS-2b (ATCC) were cultured in RPMI-1640 medium (Thermo Fisher Scientific). DBTRG-05MG (ATCC) and HEK293T (ATCC) were cultured in Dulbecco's Modified Eagle Medium (DMEM) medium (Thermo Fisher Scientific). SKOV3 (ATCC) was cultured in McCoy’s 5A medium (Sigma-Aldrich). Human umbilical vein endothelial cells (HUVECs) were cultured in F-12K medium (ATCC) supplemented with 0.1 mg/mL heparin, 50 µg/mL endothelial cell growth supplement (ECGM, BD Biosciences), and 10% FBS. The Luc-expressing human glioblastoma (GBM) cell line U87 MG-Luc2 (ATCC) and SVGp12 (ATCC) were cultured in Eagle’s minimal essential medium (Thermo Fisher Scientific).

### Bioinformatics analysis

Expression of HLA-G mRNA in tumor tissues was analyzed using web-based tools in the GEPIA (http://gepia.cancer-pku.cn/) databases. Briefly, the desired gene (*HLA-G*) was searched, survival analysis was performed for all tumor types, and expression levels were plotted as boxplots using the software on the website (http://gepia.cancer-pku.cn/).

### Expansion of primary NK cells

Human NK cells were isolated from peripheral blood mononuclear cells (PBMC) (Stem Cell Technologies) by using EasySep Human NK Cell Isolation Kit (Stem Cell Technologies). In brief, 5×10^7^ PBMCs were incubated with 50 µL Human NK Cell Isolation Cocktail in a total 2 mL PBS containing 1% FBS for 5 min at room temperature, then added to 50 µL Dextran RapidSpheres and then immediately gently mixed. After 3 min incubation in the magnet, the enriched cell suspensions were centrifuged and harvested. The isolated NK cells were expanded by using NK Cell Activation/Expansion Kit (Miltenyi Biotec). A 5×10^6^ isolated NK cell was included in 15 mL of X-VIVO15 medium (Lonza) containing mixed biotinylated CD335 and CD2 antibodies and Anti-Biotin MACSiBead Particles (bead-to-cell as 1:2) supplemented with 500 IU/mL interleukin-2 (Peprotech) and 10% platelet-rich plasma. The medium was refreshed every 2–3 days for a total of 3 weeks’ culture. After that, the expanded cells were counted, and its purity and phenotypical markers were determined by flow cytometry using specific antibodies.

### Construction of the lentiviral vector

The HuScL-2 Human Single-Chain Antibody Library (Creative Biolabs) was screened; after four rounds, 40 clones from the fourth eluate were selected for analysis in ELISA using monoclonal phages. The highest affinity clone was selected and its single-chain variable fragment (scFv) was used to build the anti-HLA-G CAR construct by synthesizing DNA corresponding to the leader peptide sequence 5′-ATGGCCCTCCCTGTCACCGCCCTGCTGCTTCCGCTGGCTCTTCTGCTCCACGCCGCTCGGCCC-3′. This was fused to the anti-HLA-G scFv sequence, followed by the DNA sequence encoding the transmembrane and cytosolic domains of KIR2DS4 (amino acids 246–304), the P2A (porcine teschovirus-1 2A) sequence (amino acids 1–19), the full-length DAP-12 (DNAX-activating protein of molecular mass 12 kD) sequence (GenBank: NM_003332.3), and inducible caspase-9 (iC9) (FK506-binding protein 12 (amino acids 1–108) fused to caspase-9 (amino acids 135–416)). The insert was cloned into the pCAR-(puroless) lentiviral vector (Creative Biolabs) via the EcoRI/XbaI restriction sites.

### Lentiviral transduction

The α-HLA-G CAR plasmid, pMD2G, and psPAX2 were transfected into HEK293T cells at a ratio of 5:3:1 using Lipofectamine 3000 (Invitrogen). The supernatant was collected after 48 hours. Then, the primary NK cells (5×10^5^) were pretreated for 30 min with BX795 (8 µM), washed with PBS, resuspended in Opti-MEM (500 µL), and then added to the virus-containing medium (multiplicity of infection=3) with 50 µg/mL protamine sulfate and 8 µM BX795. On the following day, the medium was replaced with X-VIVO15 complete medium containing mixed CD335/CD2 MACSiBead Particles. After 1 week, the transduction rate, phenotypical markers, and purity were determined by flow cytometry using protein L staining and specific antibodies, respectively.

### Protein L staining and fluorescence-activated cell sorting (FACS) analysis of purity and phenotypical markers in NK cells

Expression of the anti-HLA-G fragment on the NK cell surface was determined using protein L staining, as described previously.^34^ Briefly, HLA-G CAR-transduced primary NK cells were harvested, washed, resuspended in PBS containing 4% FBS and protein L (1 µg), incubated for 45 min, washed three times, resuspended for 45 min in PBS containing Alexa Fluor 594-conjugated streptavidin (5 µg/mL, Thermo Fisher Scientific) and FITC-conjugated anti-CD56 antibody, and washed again three times. Protein L and CD56 dual-positive cells were examined by flow cytometry, with parental NK cells used as background controls.

### Cytotoxic killing and cytotoxicity assays

Effector cells and target cells were cocultured for 24–72 hour at 37°C with effector cells at effector:target (E:T) cell ratios of 0.1:1.0 to 10:1, with or without pretreatment for 48 hours with Dox (50 nM), TMZ (80 µM), Gem (10 µM), or CBP (20 µM). To detect cell apoptosis, cocultured cells were stained with an antiannexin V antibody and propidium iodide (PI) using BD Annexin V: FITC Apoptosis Detection Kit I (BD Biosciences). Target cells were determined based on size; cells showing the presence of granules and annexin V^high^ or PI^high^ staining were considered ‘killed cells’. The cell killing rates are presented as a percentage of the total cell count. To determine the cytotoxicity induced by chemotherapeutic agents, the cells were treated with or without titrated Dox, TMZ, Gem, or CBP for 24 and 48 hours, then the cell viability was measured by PI staining and flow cytometry analysis.

### Cytokine assays

Human cytokine granzyme B, perforin, interferon gamma (IFN-γ), and tumor necrosis factor alpha (TNF-α) were measured using commercial ELISA kits (Thermo Fisher Scientific).

### Immunoblot analysis

Cell platelets were harvested and resuspended in a protease inhibitor cocktail containing PRO-PREP protein extraction buffer (iNtRON) and vigorously shaken at 4℃ for 15 min. The supernatants were collected, and the protein concentrations were measured. Sample lysates (50 µg) were subjected to electrophoresis in sodium dodecyl sulfate–polyacrylamide gel electrophoresis, transferred onto polyvinylidene difluoride membranes, blocked at 4°C for 1 hour with tris-buffered saline with Tween (TBST)/5% bovine serum albumin (BSA), and then incubated overnight at 4℃ with primary antibodies. Next, membranes were washed four times with TBST, incubated at 25°C±1°C for 2 hour with horseradish peroxidase-conjugated goat antimouse or rabbit IgG (Thermo Fisher Scientific) secondary antibodies, washed again, and incubated for 1 min with SuperSignal West Pico ECL reagent (Pierce Biotechnology). Chemiluminescence was detected using the ChemiDoc Imaging System (Bio-Rad Laboratories), with β-actin as the internal control.

### Flow cytometry

Briefly, cells cultured with or without chemotherapeutic agents or cocultured with NK cells were harvested, washed with PBS, and stained at 4°C for 45 min with fluorophore-conjugated human anti-HLA-G antibodies (Abcam). After washing twice with PBS, cells were analyzed using a Cytomics FC500 flow cytometer (Beckman Coulter) using the FL1 or FL2 channel. Cell surface expression of HLA-G on tumor cells and the expression of HLA-G, Fas ligand (FasL), TRAIL, and CD107a by NK cells in the different treatment groups were determined by comparison with the mean fluorescence intensity of untreated control cells (set to 100%). To determine the purity and phenotypical markers of NK cell products, the NK cells were stained with FITC-conjugated anti-CD45 antibody combined anti-CD56, CD16, CD3, CD19, CD11b, or CD66b antibodies, or with FITC-conjugated CD56 combined CD94, NKG2D, Nkp44, Nkp46, and Nkp30 antibodies for 45 min, respectively. Thereafter, the cells were washed, and the cell frequency in CD45-positive cells and the expression of phenotypical markers in CD56 positive cells were analyzed.

### Transient expression and stable clones

Small interfering RNAs (siRNAs) targeting human transporter associated with antigen processing 1 (TAP-1) and SPP (Santa Cruz Biotechnology) were transfected into MDA-MB-231 and DBTRG-05MG cells using the INTERFERin siRNA transfection reagent (Polyplus). After the indicated times, cells were treated with Dox or TMZ for the indicated times and subjected to other assays. To generate stable clones, HLA-G small hairpin RNA plasmids (Santa Cruz Biotechnology) and HLA-G and DNMT1 expression plasmids (Origene) were transfected for 48 hours into MDA-MB-231 and AsPC-1 cells using Lipofectamine 3000. Transfected cells were selected for 21 days using puromycin, and a single-cell clone was expanded and harvested to determine knockdown efficiency by immunoblotting with specific antibodies.

### Immunocytochemistry

Tumor cells or primary human hepatocytes (1×10^5^) were seeded on coverslips in a six-well plate and incubated overnight. After the indicated treatments, cells were fixed in 1% paraformaldehyde, washed with PBS, permeabilized using 0.1% Triton X-100 in PBS containing 0.5% BSA for 30 min, blocked with 2% BSA, and incubated with specific antibodies in 2% BSA/PBS containing 0.05% Tween-20 (phosphate buffered saline with Tween (PBST)). After washing, the cells were incubated with fluorescein-conjugated secondary antibodies, washed with PBST, and mounted using a water-based mounting medium containing an antifade agent and DAPI (4',6-diamidino-2-phenylindole). Images were analyzed under a Leica TCS SP8 X confocal microscope (Leica).

### DNA extraction and bisufate conversion

A DNeasy Blood and Tissue Kit (Qiagen) was used to extract DNA. Briefly, cell samples were lysed with proteinase K and loaded onto a DNeasy Mini spin column. The bound DNA was then eluted in elution buffer. The bisulfite reaction mixture was prepared in PCR tubes (200 µL) containing DNA (2 µg), bisulfate solution, and DNA Protect Buffer from the EpiTect Fast DNA Bisulfite Kit (Qiagen), followed by initialisation of the thermal cycling protocol. Then the samples were subjected to the spin column and centrifuged, and the bisulfate-converted DNA was eluted in pure water.

### TAP-1 promoter methylation-specific PCR (MSP) assay

The *TAP-1* promoter MSP assay was performed as previously described.^35^ Briefly, each reaction mixture comprised HotStarTaq Master Mix (Qiagen), forward and reverse primers targeting the *TAP-1* promoter, RNase‐free water, and bisulfate-converted DNA. The MSP primer sequences are as follows: methylated forward, 5′-TTTTTTAAATGGTTGAGTTTTTCGT-3′, and reverse, 5′-TAAAACCTAAAACTCCGAATACCG-3′; unmethylated forward, 5′-TTTTTTAAATGGTTGAGTTTTTTGT-3′, and reverse, 5′-AAAACCTAAAACTCCAAATACCACC-3′. Reactions were started by heating to 95°C for 5 min, followed by 35 cycles of 95°C for 45 s, 57°C for 30 s and 72°C for 30 s, and a final extension for 10 min at 72°C. CpGenome Universal Methylated DNA (Sigma-Aldrich) and CpGenome Universal Unmethylated DNA (Sigma-Aldrich) kits were used as positive controls for methylated and unmethylated DNAs, respectively, whereas Ultrapure distilled water (Invitrogen) served as the negative control. The MSP products were separated on a 2% agarose gel and visualized using ethidium bromide staining with ultraviolet light transillumination.

### MDA-MB-231 and U87 orthotopic xenograft mouse models

Six to 8 weeks old NOD/SCID gamma (NSG) mice were purchased from The Jackson Laboratory. Female mice were used for the xenograft triple-negative breast cancer (TNBC) tumor model, whereas male mice were used for the xenograft GBM tumor model. For TNBC tumors, Luc^+^ MDA-MB-231 cells were resuspended in PBS containing Matrigel, then the cells (1×10^6^/100 µL) were injected subcutaneously into the left fourth mammary gland of female mice. For GBM tumors, male mice were anesthetized, and a burr hole was drilled into the skull (1 mm posterior to bregma and +2 mm mediolateral from the midline). Luc^+^ U87 cells (1×10^6^/5 µL) in PBS were delivered intracranially using a Hamilton syringe (Reno) at a depth of 3 mm with 5 min intervals. After the delivery, mice were allowed to rest for at least 30 min before the wound was closed. Seven days after implantation, each mouse was infused with thawed α-HLA-G CAR-NK cells (1.5×10^7^/100 µL PBS) via tail vein injection, followed by 3 weekly infusions (5×10^6^) for the next 3 weeks. Tumor growth was monitored weekly via bioluminescence imaging using the in vivo imaging system (IVIS) (PerkinElmer). Then, on the day before NK cell infusion, the mice received intravenous Dox (0.5 mg/kg) or oral gavage TMZ (3 mg/kg). On the indicated days, mice were euthanized, and the tumors were harvested, measured, and photographed.

### Immunohistochemistry

Tumor samples were fixed in 10% formaldehyde, embedded in paraffin, and sectioned (3 µm thick). Normal tissue arrays were purchased from Pantomics, and slides were heated in 1 mM EDTA buffer for 30 min and then incubated with H_2_O_2_ for 15 min for antigen retrieval. Tissue sections were incubated for 18 hours at 4°C with primary antibodies (antihuman HLA-G (4H84)). Then, sections were incubated with biotin-conjugated antimouse IgG secondary antibody, followed by incubation with a polymer for 10 min at 25°C±1°C. Finally, sections were counterstained with haematoxylin. Quantification was performed by two pathologists (C-IJ and PC) under an optical microscope (Eclipse 80i, Nikon) at ×400 magnification. The expressions of HLA-G in three non-overlapping high-power fields were counted. The HLA-G H score was determined by the following formula: 3×percentage of strong membrane staining+2×percentage of moderate membrane staining+percentage of weak membrane staining, giving a range of 0–300.^36^

### Activation of the safety switch

For in vitro evaluation, the chemical inducer of dimerisation compound AP20187 was added at different concentrations to NK cell cultures, and apoptosis induction was evaluated after 24, 48, and 72 hours by PI staining using flow cytometry analysis. For in vivo evaluation, MDA-MB-231 tumor-bearing mice were infused with thawed anti-HLA-G CAR-NK cells (1.5×10^7^) and treated with or without AP20187 (5 mg/kg) via the tail vein injection the following day. After 7 days, luminescent signals were recorded and quantified to determine residual antitumor activity, and the mice were euthanized. The persistence of anti-HLA-G CAR-NK cells was measured by flow cytometry of CD56/protein L-stained splenocytes.

### Statistical analysis

Data analysis was performed with SigmaPlot V.11.0 and GraphPad Prism software. Differences in tumor growth curves were determined using analysis of variance, and cytokine assay data were assessed using Student’s t-test. Survival and tumor growth were analyzed using the Kaplan-Meier method and the log-rank test. P values of <0.05 were considered statistically significant.

## Results

### HLA-G is expressed widely by various malignant cells but is barely detectable in nearly normal cells

**Figure 1:**
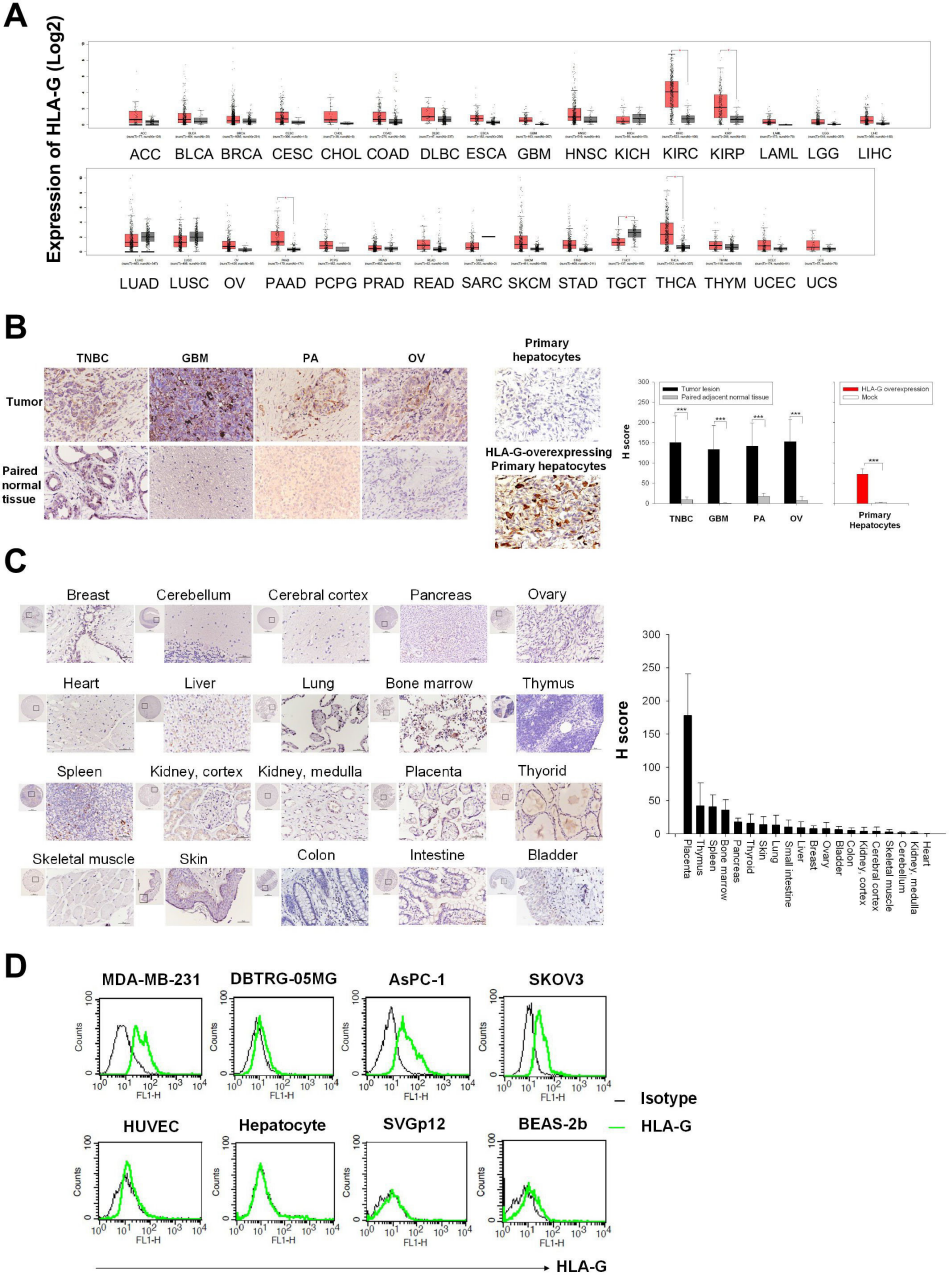
Expression of HLA-G in tumor and normal tissues and cells. (A) Expression of mRNA encoding HLA-G in matched tumor and normal tissues was extracted from the GEPIA databases and presented as boxplots. (B) HLA-G protein expression in tumor lesions (upper panel) and matched healthy tissues (lower panel) from patients with TNBC, GBM, PA, and OV cancers, as determined by immunohistochemical analysis with anti-HLA-G antibodies. The primary human hepatocyte formalin-fixed, paraffin-embedded (FFPE) cell pellets with or without HLA-G-expressing plasmid transfection as staining controls (middle panel). (C) Representative micrographs of HLA-G proteins on a normal tissue microarray. Tissue sections were stained with anti-HLA-G antibodies. Scale bars, 50 µm. Original magnification: ×400. The expression of HLA-G on cell membrane was counted and presented as H score. (D) Cell surface expression of HLA-G by solid tumor cell lines and immortalized non-malignant cell lines. MDA-MB-231, DBTRG-05MG, AsPC-1, SKOV3, HUVEC, SVGp12, and BEAS-2b cells and primary hepatocytes, were stained with FITC-conjugated anti-HLA-G antibodies or with isotype-matched control antibodies, and flow cytometry analysis was conducted using the FL1 channel. The results are presented as histograms. GBM, glioblastoma; HLA-G, human leukocyte antigen G; HUVEC, human umbilical vein endothelial cell; OV, ovarian cancer; PA, pancreatic cancer; TNBC, triple-negative breast cancer.

First, we used the GEPIA databases to ascertain HLA-G expression by various tumors and matched adjacent healthy tissues. Generally, the median HLA-G mRNA expression was elevated 2-fold to 10-fold compared with matched healthy tissues, but the differences only reached significance in kidney renal clear cell carcinoma, kidney renal papillary cell carcinoma, pancreatic ductal adenocarcinoma, and thyroid cancer. They have a significantly higher level of HLA-G mRNA expression when compared with normal tissues. Nevertheless, there is a subpopulation in most tumor types with elevated HLA-G mRNA expression. Therefore, patients with such might be identified by HLA-G-specific immunotherapy screening methods such as immunohistochemistry staining. Thus, we investigated the expression level of HLA-G in tumor and normal tissues by immunohistochemistry (IHC) staining and quantified the H score. Histological sections of TNBC, GBM, pancreatic cancer (PA), and ovarian cancer (OV) showed strong cell membrane and cytosolic staining of HLA-G, which were absent from paired peritumoral healthy tissues. The H scores of HLA-G in these tumor sections (average 133~152) were significantly higher than those in their paired adjacent tissues (average 1.2~17) (figure 1B). In addition, some stoma cells such as mononuclear cells, fibroblasts, and endothelium cells showed weak to strong positive staining in their cell membrane and/or cytoplasm. IHC staining of normal tissue array chips revealed that HLA-G was not expressed in most vital organs. However, it was expressed by some macrophages and megakaryocytes in thymus, spleen, and bone marrow. A few pancreas, thyroid, and lung tissues expressed low levels of HLA-G (average H score 14~38), whereas other tissues barely had detectable levels except for the well-characterized HLA-G-expressing placental cytotrophoblasts (figure 1C). A similar phenotype was noted in human solid tumor cell lines MDA-MB-231, DBTRG-05MG, AsPC-1, and SKOV3, and in normal primary cell cultures or transformed normal cell lines (figure 1D).

### Engineered HLA-G-specific CAR-NK cells exhibit antitumor activity in vitro

**Figure 2:**
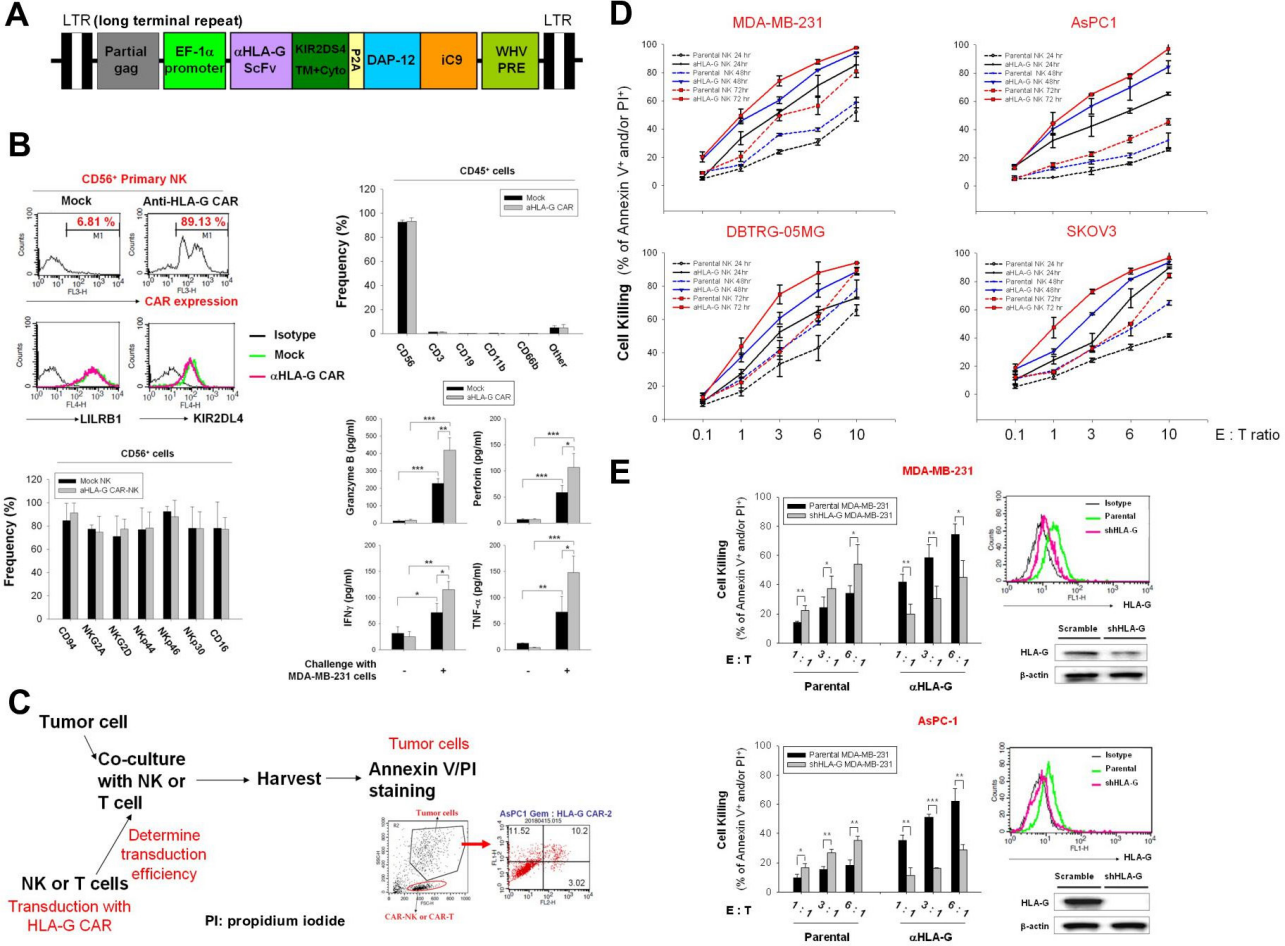
Cytotoxic killing of multiple solid tumor cell lines by anti-HLA-G CAR-NK cells. (A) Lentiviral vector map of the DAP12-based anti-HLA-G CAR construct. Schematic representation of the anti-HLA-G antibody scFv fragment linked with the transmembrane and intracellular domain of KIR2DS4 following self-cleavage peptide P2A and full-length DAP-12, fused to the suicide iC9 protein. (B) Purity, transduction efficiency and phenotypical analysis of anti-HLA-G CAR-NK cells. NK cells were transduced (or not) with anti-HLA-G CAR lentiviral particles, and expression of anti-HLA-G CAR was determined by protein L staining (upper left panel). Expression of LILRB1 and KIR2DL4 in CD56-positive cells (upper right panel), the populations of CD56, CD16, CD3, CD19, CD11b, and CD66b in CD45-positive cells (middle left panel), the frequency of CD94, NKG2D, Nkp44, Nkp46, and Nkp30 in CD56-positive cells (middle right panel), and the expression of CD56 and CD16 were analyzed by flow cytometry using specific fluorescent-conjugated antibodies (bottom left panel). The secretion of granzyme B, perforin, IFN-γ, and TNF-α form NK cells with or without MDA-MB-231 cells challenge (E:T as 1:1) were measured by ELISA assays. (C) Schematic showing the gating strategy used to determine tumor cell death (PI/annexin V staining) triggered by anti-HLA-G CAR-NK cells. (D) MDA-MB-231, DBTRG-05MG, AsPC-1, and SKOV3 cells were incubated with parental or anti-HLA-G CAR-transduced NK cells for 24, 48, and 72 hours at E:T ratios of 0.1:1.0, 1:1, 1:3, 1:6, and 1:10. (E) Characterization of stable HLA-G knockdown AsPC-1 and MDA-MB-231 cell lines by flow cytometry (upper right) and immunoblotting (lower right) with specific antibodies; the corresponding HLA-G CAR-NK-induced cytotoxicity for 48 hours at E:T ratios of 1:1, 1:3, and 1:6 was determined by flow cytometry using PI/annexin V staining (left). Tumor cell death was determined by flow cytometry after PI/annexin V staining. Data are expressed as the mean±SEM of ≥3 independent experiments (*p<0.05, **p<0.01, ***p<0.001). CAR, chimeric antigen receptor; E:T, effector:target; HLA-G, human leukocyte antigen G; IFN-γ, interferon gamma; NK, natural killer; scFv, single-chain variable fragment; TNF-α, tumor necrosis factor alpha.

The engineered scFv derived from an anti-HLA-G antibody comprised the following sequences: the transmembrane and intracellular domains of KIR2DS4, full-length DAP12, and iC9; HLA-G scFv-KIR2DS4 and DAP12-iC9 were separated by the P2A sequence. This construct was then cloned into a lentiviral vector (figure 2A). Staining for protein L indicated that the transduction efficiency of the anti-HLA-G CAR vector into NK cells was approximately 80%, and the expression levels of LILRB1 and KIR2DL4 were not modulated (figure 2B, upper left panel). The frequency of CD45-positive, CD56-positive, CD3-positive, CD19-positive, CD11b-positive, and CD66b-positive cells in the final NK products are shown in the upper right panel of figure 2B. The CD3^+^ cells were approximately 1.23% in mock NK and 1.36% in CAR-NK, and the frequency of CD56^+^ cells was over 92% in these cell products. Phenotypical analysis after CAR transduction revealed that the frequency of CD94, NKG2A, NKG2D, NKp44, NKp46, and CD16 demonstrated no difference between the CD56^+^ parental and CAR-NK cells (figure 2B, bottom left panel). In addition, these NK cell products showed increased granzyme B, perforin, IFN-γ, and TNF-α secretion on challenge with MDA-MB-231 cells; and anti-HLA-G CAR-transducing NK cells showed additional release of these cytotoxic molecules and cytokines (figure 2B, bottom right panel). These results suggested that anti-HLA-G CAR-NK cells are effector NK cells with adequate purity and maintained characteristics similar to those of the parental NK cells.

Next, we used MDA-MB-231, DBTRG-05MG, AsPC-1, and SKOV3 cells as in vitro models to test the antitumor activity of HLA-G CAR-NK cells. Figure 2C illustrates the protocol and gating strategy used to determine cytotoxic efficacy. Anti-HLA-G CAR-NK cells were more cytotoxic than the parental control NK cells; at equivalent E:T ratios and in a time and increased NK cell titration manner (figure 2D). We used stable HLA-G knockdown MDA-MB-231 and AsPC-1 cells to confirm that the increased killing ability of HLA-G CAR-NK cells was dependent on the expression of HLA-G, whereas these HLA-G knockdown cells were more sensitive to parental NK cell-mediated cytotoxicity (figure 2E). Taken together, the results suggest that HLA-G CAR increases the cytotoxic activity of NK cells against a variety of solid tumor cells, and that this cytotoxicity is dependent on HLA-G.

### HLA-G CAR converts inhibitory signals into activation signals to trigger tumor cell killing via downregulation of phosphor-SHP-1 and upregulation of phosphor-Syk/Zap70

**Figure 3:**
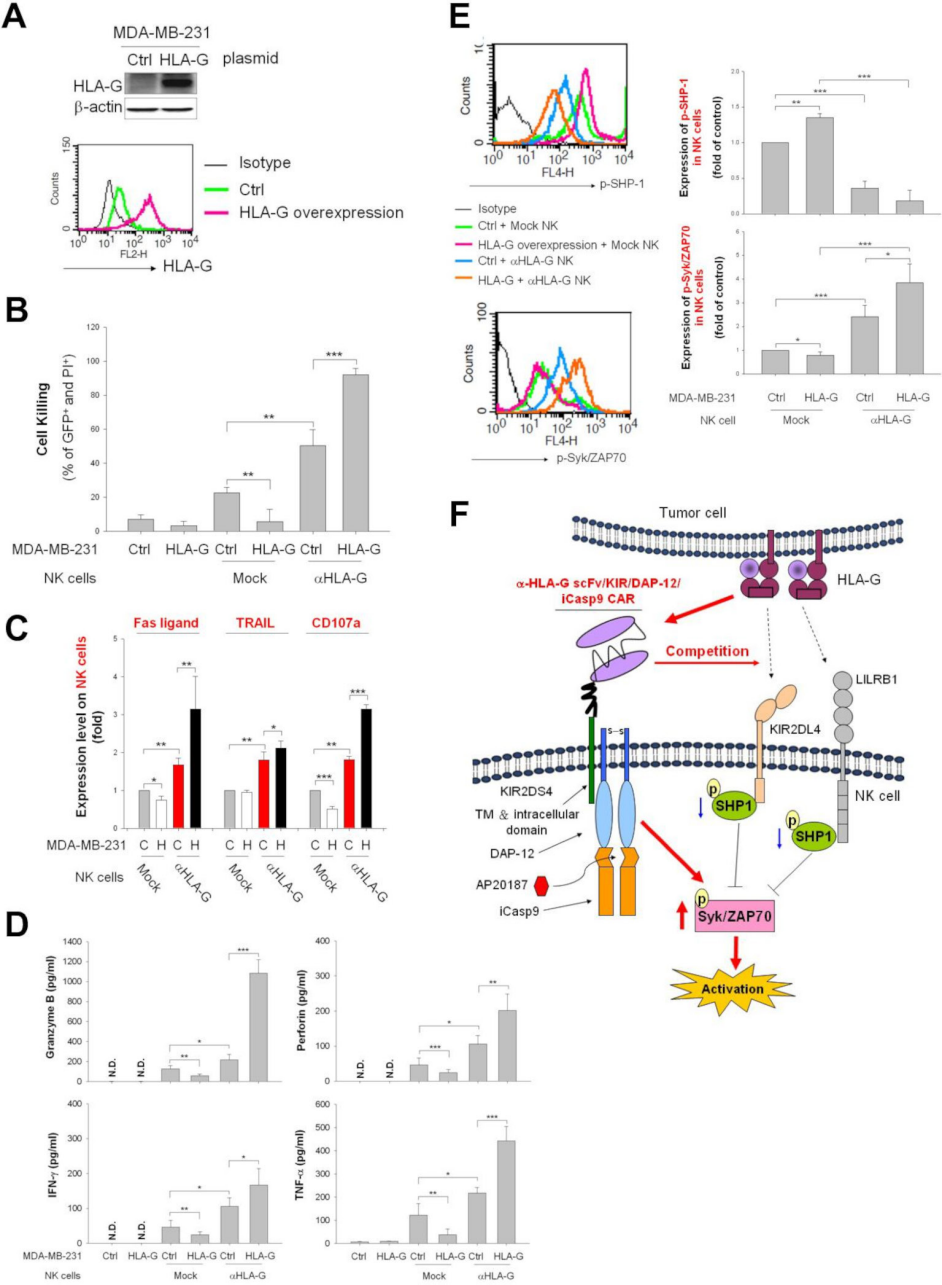
Anti-HLA-G CAR construct switches tumorous HLA-G signaling in NK cells from inhibitory to activating. (A) Overexpression of HLA-G in MDA-MB-231 cells. HLA-G levels in control and HLA-G-expressing vector-transfected MDA-MB-231 cells were determined by western blot analysis and flow cytometry using specific antibodies (see the histogram overlays). (B–D) Control and HLA-G-overexpressing MDA-MB-231 cells were cocultured with anti-HLA-G CAR-NK or mock-transfected NK cells at an E:T ratio of 1:1. After 48 hours of coculture, the cell-killing rate (B), the expression of FasL, TRAIL, and CD107a (C), and the secretion of granzyme B, perforin, IFN-γ, and TNF-α (D) of NK cells was measured by flow cytometry after PI/annexin V staining and staining with specific antibodies, respectively. (E) NK cells were harvested after 1 hour of coculture with parental or HLA-G-overexpressing MDA-MB-231 cells, and the expression of phosphorylated SHP-1 and Syk/ZAP70 were determined by intracellular staining using specific antibodies then analyzed by flow cytometry. (F) Molecular interactions between the HLA-G on tumor cells, endogenous HLA-G receptors LILRB1, and KIR2DL4, and the anti-HLA-G CAR construct on engineered NK cells. Data are expressed as the mean±SEM of ≥3 independent experiments (*p<0.05, **p<0.01, ***p<0.001). CAR, chimeric antigen receptor; Ctrl, control; E:T, effector:target; HLA-G, human leukocyte antigen G; IFN-γ, interferon gamma; NK, natural killer; TNF-α, tumor necrosis factor alpha.

Next, we generated a stable HLA-G-overexpressing MDA-MB-231 clone to evaluate whether CAR-NK cells override inhibitory HLA-G signals to activate antitumor responses. As shown in figure 3A, expression of total and membrane-bound HLA-G was measured by immunoblotting and flow cytometry. HLA-G-overexpressing MDA-MB-231 cells were resistant to mock NK-induced cytolysis but were sensitive to cytolysis by HLA-G CAR-NK cells (figure 3B). This sensitivity corresponded with the expression pattern of FasL, TRAIL, and CD107a on NK cells (figure 3C) and the secretion profiles of granzyme B, perforin, IFN-γ, and TNF-α (figure 3D). The anti-HLA-G scFv component may compete with LILRB1 and KIR2D4, both of which are expressed by NK cells, to hijack tumor HLA-G and activate NK cell cytotoxicity by downregulating phosphor-SHP-1 and upregulating phosphor-Syk/Zap70 (thereby converting inhibitory signals to activating signals) (figure 3E). These results suggest that HLA-G CAR-NK cells can switch off immunosuppressive signals mediated by tumor cell-expressed HLA-G to activate cytotoxic NK killing responses (schema in figure 3F).

### Chemotherapy induces accumulation of cell HLA-G on the surface of tumor cells but not on non-malignant cells

**Figure 4:**
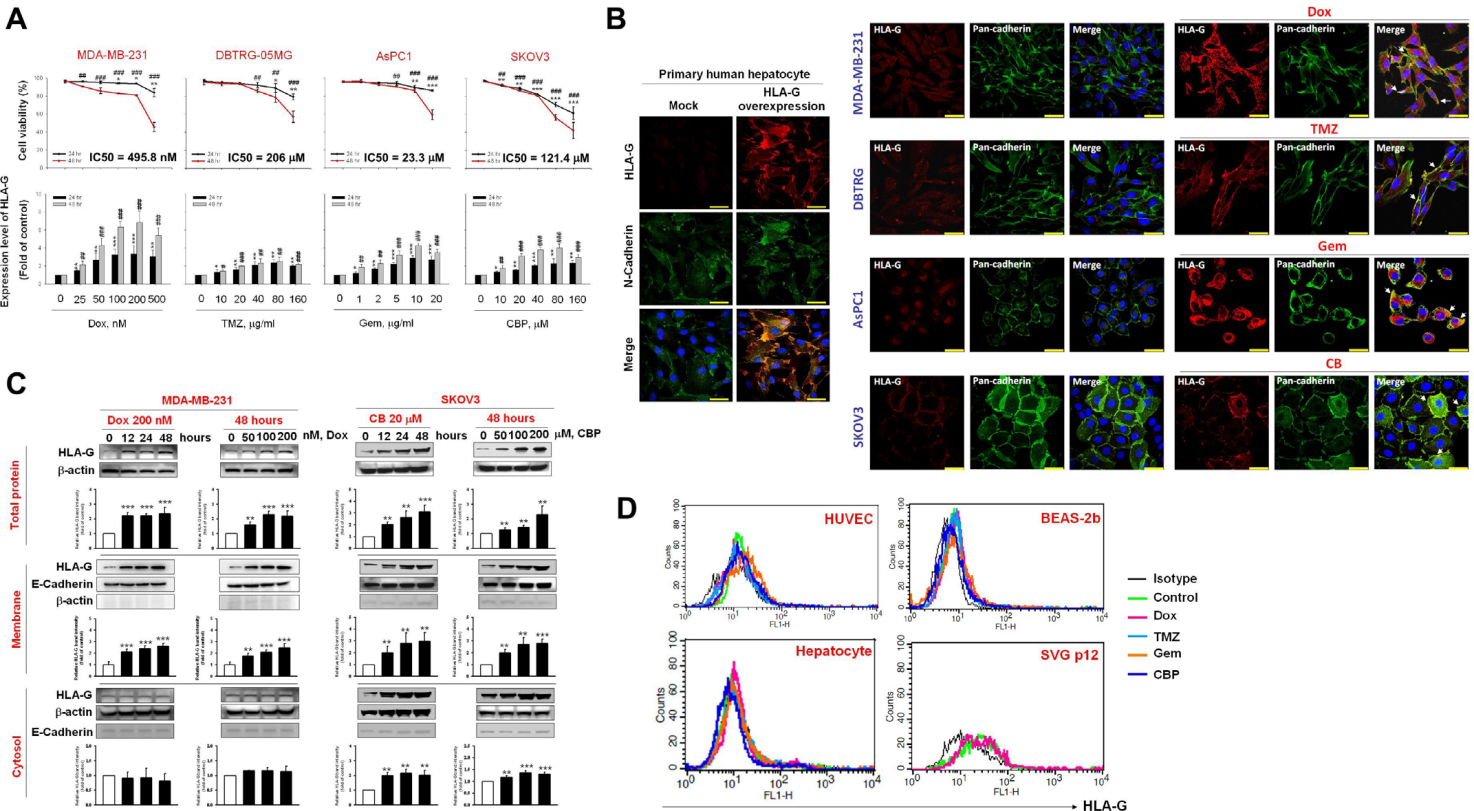
Chemotherapeutic agents upregulate membrane expression of HLA-G on tumor cell lines but not normal or normal-transformed cell lines. (A–C) Tumor cell surface expression of HLA-G after treatment with chemotherapeutic agents. MDA-MB-231, DBTRG-05MG, AsPC-1, and SKOV3 cells were treated for 48 hours with 200 nM Dox, 80 µg/mL TMZ, 20 µg/mL Gem, or 20 µM CBP, respectively. Cells were collected and analyzed to assess subcellular localisation of HLA-G and pan-cadherin using immunocytochemistry and confocal microscopy (A); scale bars, 37 µm. Cell surface expression of HLA-G is shown in the histogram overlays (B). Expression of HLA-G, E-cadherin, and β-actin in the cytosol and membrane fractions, as assessed by immunoblotting with specific antibodies. The primary human hepatocytes transfected with or without HLA-G expression plasmid as staining controls (C). (D) Expression of HLA-G expression on normal or normal-transformed cell lines following chemotherapy. HUVECs, primary hepatocytes, BEAS-2b cells, and SVGp12 cells were treated for 48 hours with or without 200 nM Dox, 80 µg/mL TMZ, 20 µg/mL Gem, and 20 µM CBP, respectively. Cells were harvested and HLA-G expression was analyzed using flow cytometry after staining with specific antibodies (see the histogram overlays). Data are expressed as the mean±SEM of ≥3 independent experiments (*p<0.05, **p<0.01, ***p<0.001). HLA-G, human leukocyte antigen G; HUVEC, human umbilical vein endothelial cell.

Reports suggest that chemotherapy improves the efficacy of CAR-T cell therapy by inhibiting suppressive cells or by prolonging the persistence of CAR-T cells.^37^ However, no study has used HLA-G as a chemotherapy-inducible target for CAR cell therapy. We found that chemotherapeutic agents traditionally used to treat TNBC, GBM, PA, and OV (ie, Dox, TMZ, Gem, and CBP) decreased cell viability and enhanced membranous HLA-G expression in a time-dependent and dose-dependent manner (figure 4A). Immunocytochemistry showed that Dox, TMZ, Gem, and CBP promoted translocalisation of HLA-G on the membrane in MDA-MB-231, DBTRG-05MG, AsPC-1, and SKOV3 cells, respectively (figure 4B). Immunoblotting confirmed that Dox and CBP increased expression of HLA-G in cell membrane fractions from MDA-MB231 and SKOV-3 cells, respectively (figure 4C). In addition, expression of membrane-bound HLA-G by HUVECs, primary human hepatocytes, bone marrow cells, immortalized human bronchial epithelial cells (BEAS-2b), or immortalized human fetal glial cells (SVGp12) was unchanged (ie, remained very low or absent) after exposure to these chemotherapeutic agents (figure 4D).

### Chemotherapeutic agents reduce expression of DNMT1 and demethylation of the TAP-1 promoter to trigger translocation of HLA-G

**Figure 5:**
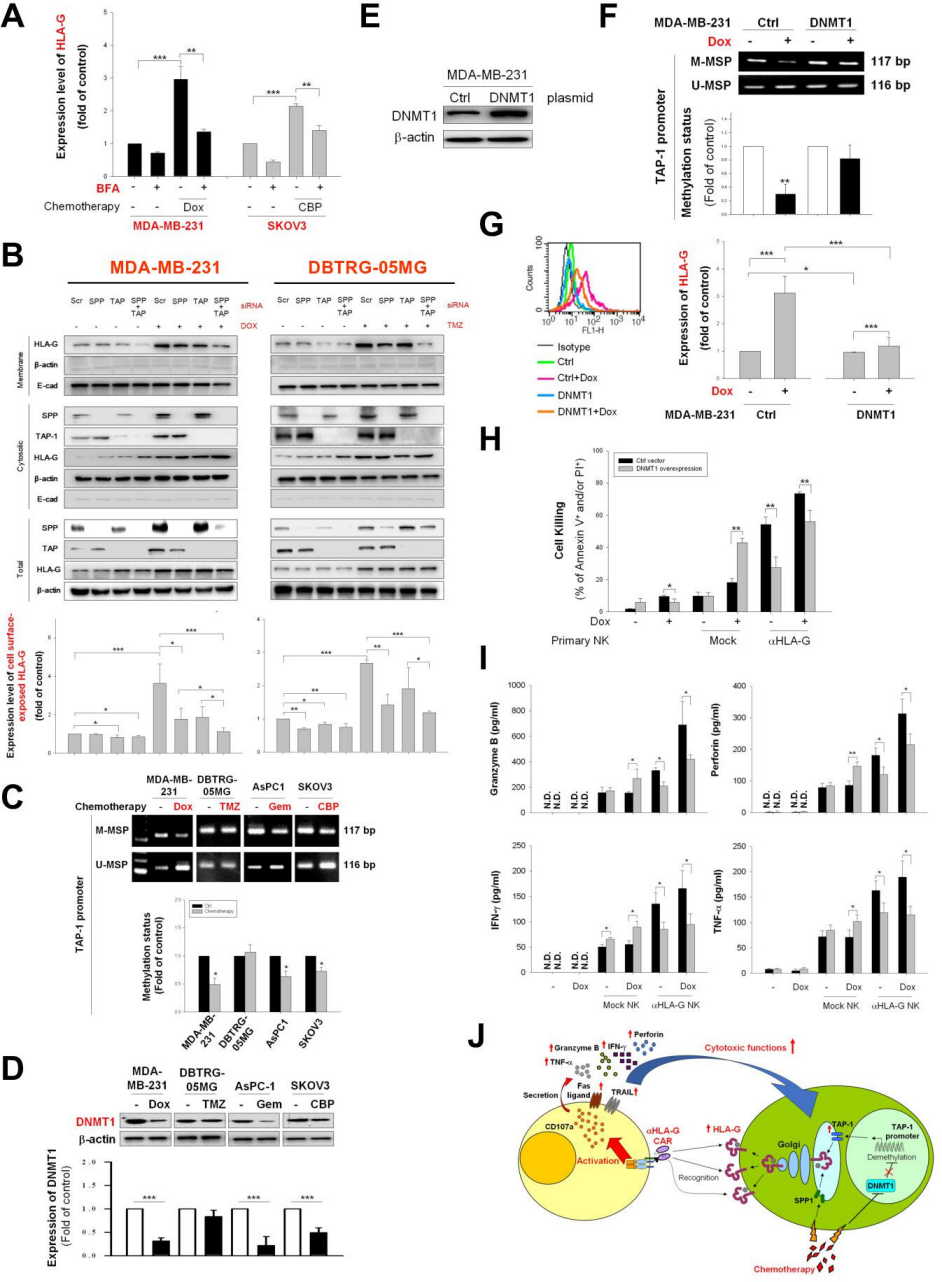
Genetic/epigenetic regulation of TAP-1 and genetic expression of SPP in tumor cells treated with chemotherapeutic agents affect cell surface expression of HLA-G via altered Golgi trafficking. (A) MDA-MB-231 and SKOV3 cells were treated (or not) for 24 hours with 200 nM Dox or 20 µM CBP, followed by incubation with 2 µg/mL BFA for an additional 16 hours. Cell surface expression of HLA-G was determined by flow cytometry. (B) MDA-MB-231 or DBTRG-05MG cells were transfected for 48 hours with control, TAP-1, or SPP siRNA, and then treated with 200 nM Dox or 80 µg/mL TMZ. Expression of HLA-G, TAP-1, SPP, and β-actin in the membrane and cytosolic fractions was determined by immunoblotting (upper panels). Relative cell surface expression of HLA-G was evaluated by flow cytometry (lower panel). (C) MDA-MB-231, DBTRG-05MG, AsPC-1, and SKOV3 cells were treated (or not) with 200 nM Dox, 80 µg/mL TMZ, 20 µg/mL Gem, or 20 µM CBP for 48 hours, and *TAP-1* gene promoter methylation status was analyzed by methylated-MSP and unmethylated-MSP PCR. (D) DNMT1 expression in chemotherapeutic agent-treated tumor cells. MDA-MB-231, DBTRG-05MG, AsPC-1, and SKOV3 cells were treated (or not) with 200 nM Dox, 80 µg/mL TMZ, 20 µg/mL Gem, or 20 µM CBP for 48 hours. Expression of DNMT-1 and β-actin was analyzed by immunoblotting (upper panel); relative expression (measured by western blotting) is shown in the lower panels. (E, F) Control and DNMT1-overexpressing MDA-MB-231 cells were treated (or not) with Dox (200 nM) for 48 hours, and expression of DNMT1 in control and DNMT1-overexpressing MDA-MB-231 cells was detected by immunoblotting (E). The methylation status of the *TAP-1* gene promoter was analyzed by methylated-MSP and unmethylated-MSP PCR (F). (G) Control and DNMT1-overexpressing MDA-MB-231 cells were treated (or not) with Dox (200 nM) for 48 hours, and cell surface expression of HLA-G was determined by flow cytometry after staining with specific antibodies. (H, I) Expression of DNMT1 affects sensitivity to anti-HLA-G CAR-NK. Control and DNMT-overexpressing MDA-MB-231 cells were pretreated (or not) with Dox (50 nM) for 48 hours and then incubated with anti-HLA-G CAR-NK or mock NK cells for an additional 48 hours. Cell killing by NK cells was measured by flow cytometry after PI/annexin V staining (H), the secreted granzyme B, perforin, IFN-γ, and TNF-α were detected by ELISA (I). (J) Schematic representation for the mechanism by which chemotherapy-induced increases HLA-G expression and cytokines secreted by CAR-NK cells. Data are expressed as the mean±SEM of at least three independent experiments (*p<0.05, **p<0.01, ***p<0.001). BFA, brefeldin A; CAR, chimeric antigen receptor; CBP, carboplatin; Ctrl, control; Dox, doxorubicin; Gem, gemcitabine; HLA-G, human leukocyte antigen G; IFN-γ, interferon gamma; MSP, methylation-specific PCR; NK, natural killer; SPP, signal peptide peptidase; TMZ, temozolomide; TNF-α, tumor necrosis factor alpha.

Cell surface presentation of major histocompatibility complex (MHC) class I antigens occurs through the classical proteasome TAP-1/TAP-2–peptide complex pathway, or via the alternative SPP–cleavage pathway, which is proteasome-independent when TAP-1 is deficient.^38^ Antigens are then delivered to the cell membrane via the endoplasmic reticulum and Golgi apparatus.^39^ Thus, we hypothesized that HLA-G could be transported to the cell surface via a similar system after chemotherapy.^40^ We found that blocking Golgi transportation using BFA reduced cell surface expression of HLA-G by Dox-treated MDA-MB-231 and CBP-treated SKOV3 cells (figure 5A). Knockdown of SPP or TAP-1 prevented accumulation of HLA-G on the cell surface (figure 5B, lower panel) and in the membrane fractions (figure 5B, upper panel) of TMZ-treated DBTRG-05MG cells and Dox-treated MDA-MB-231 cells, respectively. Inhibition of DNMT1 increased expression of major MHC-I^41^ through increased TAP-1 expression, mediated by decreased TAP-1 gene promoter methylation.^42^ Chemotherapeutic agents such as Dox and Gem inhibit DNMT to exert their antitumor activity.^43 44^ Therefore, we asked whether Dox, TMZ, Gem, or CBP regulate DNMT1 expression and TAP-1 promoter methylation status to regulate HLA-G translocation. Treatment of MDA-MB-231, AsPC-1, and SKOV3 cells with Dox, Gem, and CBP reduced *TAP-1* promoter methylation (figure 5C) and reduced expression of DNMT1 protein (figure 5D); however, TMZ did not modulate *TAP-1* promoter methylation or DMNT1 expression in DBTRG-05MG cells. We also generated a stable DNMT1-overexpressing MDA-MB-231 clone (figure 5E) to confirm that Dox does not cause significant demethylation of the *TAP-1* promoter (figure 5F); it only slightly increased the cell surface-exposed HLA-G (figure 5G) due to DMNT1 cannot be decreased, that also reflects to more resistant to HLA-G CAR-NK-induced cytotoxic killing when compared with control cells (figure 5H), and corresponded to the secretion patterns of granzyme B, perforin, IFN-γ and TNF-α (figure 5I). We also used the well-known DNMT1 inhibitor 5-aza^45^ to address whether DNMT1 regulates cell surface HLA-G expression of tumor cells and evaluated its effect on subsequent CAR-NK challenge. As shown in online supplemental figure 1A, the treatment of 5-aza upregulated cell surface HLA-G on MDA-MB-231, DBTRG-05MG, AsPC-1, and SKOV3 cells. 5-aza enhanced the expression of membranous HLA-G that was associated with downregulation of DNMT1 and upregulation of TAP-1 (online supplemental figure 1B). 5-aza also reduced the methylation status of *TAP-1* promoter in MDA-MB-231, DBTRG-05MG, AsPC-1, and SKOV3 cells (online supplemental figure 1C). Furthermore, pretreatment with 5-aza also sensitized MDA-MB-231 and SKOV3 cells to subsequently enhance HLA-G CAR-NK-mediated cytotoxic killing (online supplemental figure 1D). Taken together, these results indicate that chemotherapeutic agents promote translocation of HLA-G to the cancer cell surface by downregulating DMNT1 expression and *TAP-1* promoter demethylation. A diagram of the proposed mechanism underlying induction of enhanced tumor cell membrane HLA-G expression by low-dose chemotherapy, followed by targeting by HLA-G CAR-NK cells, is shown in (figure 5J).

10.1136/jitc-2021-003050.supp1 Supplementary data

10.1136/jitc-2021-003050.supp2 Supplementary data

### Low-dose chemotherapy sensitizes tumor cells to HLA-G CAR-NK cell-mediated cytotoxicity in vitro

**Figure 6:**
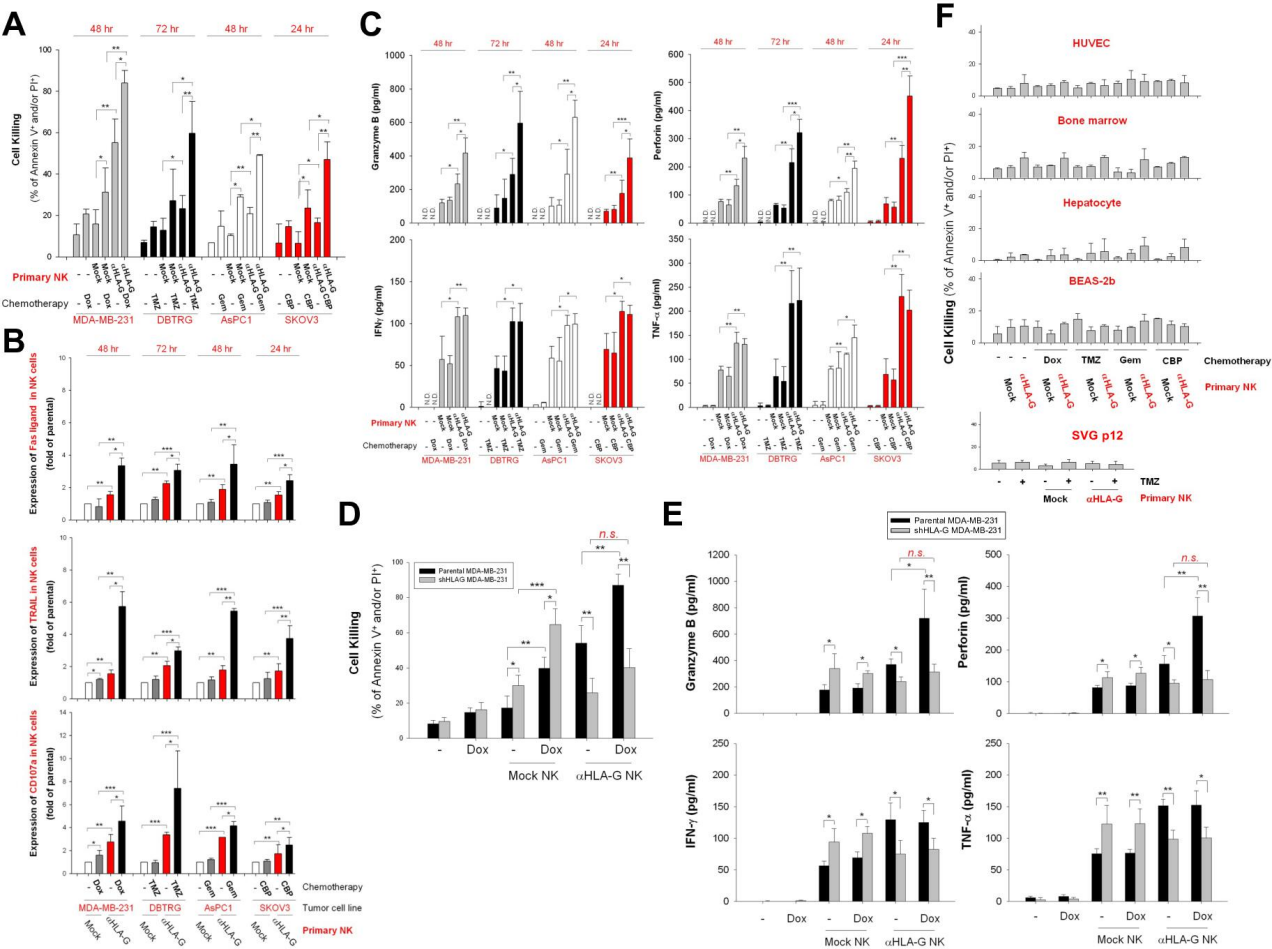
Pretreatment with chemotherapeutic agents sensitizes tumor cells but not non-malignant cells, to anti-HLA-G CAR-NK-induced cell death. (A) MDA-MB-231, DBTRG-05MG, AsPC-1, and SKOV3 cells were pretreated with 50 nM Dox, 40 µg/mL TMZ, 10 µg/mL Gem, and 10 µM CBP, respectively, for 48 hours. Subsequently, cells were incubated with mock control or HLA-G CAR-NK cells for 24, 48, or 72 hours. Tumor cell death was assessed at the indicated times by flow cytometry after PI/annexin V staining. (B) FasL, TRAIL, and CD107a expression on NK cells was detected by flow cytometry after staining with specific antibodies. (C) Granzyme B, perforin, IFN-γ, and TNF-α contents in supernatants collected from NK and tumor cell cocultures were measured by ELISA. (D, E) Cytotoxicity effects of HLA-G CAR-NK or mock NK cells against stable HLA-G knockdown MDA-MB-231 cells were determined after 48 hours of coculture by flow cytometry after staining with PI/annexin V staining (D), and the contents of granzyme B, perforin, IFN-γ, and TNF-α in supernatants were analyzed by ELISA. (F) HUVECs, primary bone marrow cells, primary hepatocytes, BEAS-2b cells, and SVGp12 cells were pretreated for 48 hours with the same dose of chemotherapeutic agents used for the aforementioned tumor cell lines, after which they were incubated for 24, 48, or 72 hours with parental or HLA-G CAR-NK cells from donors. Cell death was evaluated by flow cytometry after PI/annexin V staining. Data are expressed as the mean±SEM of ≥3 independent experiments (*p<0.05, **p<0.01, ***p<0.001). CAR, chimeric antigen receptor; CBP, carboplatin; Dox, doxorubicin; Gem, gemcitabine; HLA-G, human leukocyte antigen G; HUVEC, human umbilical vein endothelial cell; IFN-γ, interferon gamma; NK, natural killer; TMZ, temozolomide; TNF-α, tumor necrosis factor alpha.

Next, we asked whether low-dose chemotherapy-induced expression of HLA-G specifically enhances the cytotoxic effects of HLA-G CAR-NK cells. As shown in figure 6A, pretreatment of MDA-MB-231, DBTRG-05MG, AsPC-1, and SKOV3 cells with Dox, TMZ, Gem, or CBP led to a marked increase in the cytotoxic activity of HLA-G CAR-NK cells. This corresponded to increased expression of FasL, TRAIL, and CD107a (figure 6B), as well as the secretion of granzyme B and perforin but not IFN-γ and TNF-α by CAR-NK cells (figure 6C). In addition, pretreatment with low-dose Dox did not sensitize stable HLA-G knockdown MDA-MB-231 cells to HLA-G CAR-NK-induced cytotoxicity (figure 6D). Notably, HLA-G CAR-NK cells did not induce significant cell death in HUVECs, primary human hepatocytes, bone marrow cells, BEAS-2b cells, or SVGp12 cells, even after exposure to chemotherapeutic agents (figure 6E).

### Low-dose chemotherapy stimulates HLA-G CAR-NK cell cytotoxic activity against solid tumors in vivo

**Figure 7:**
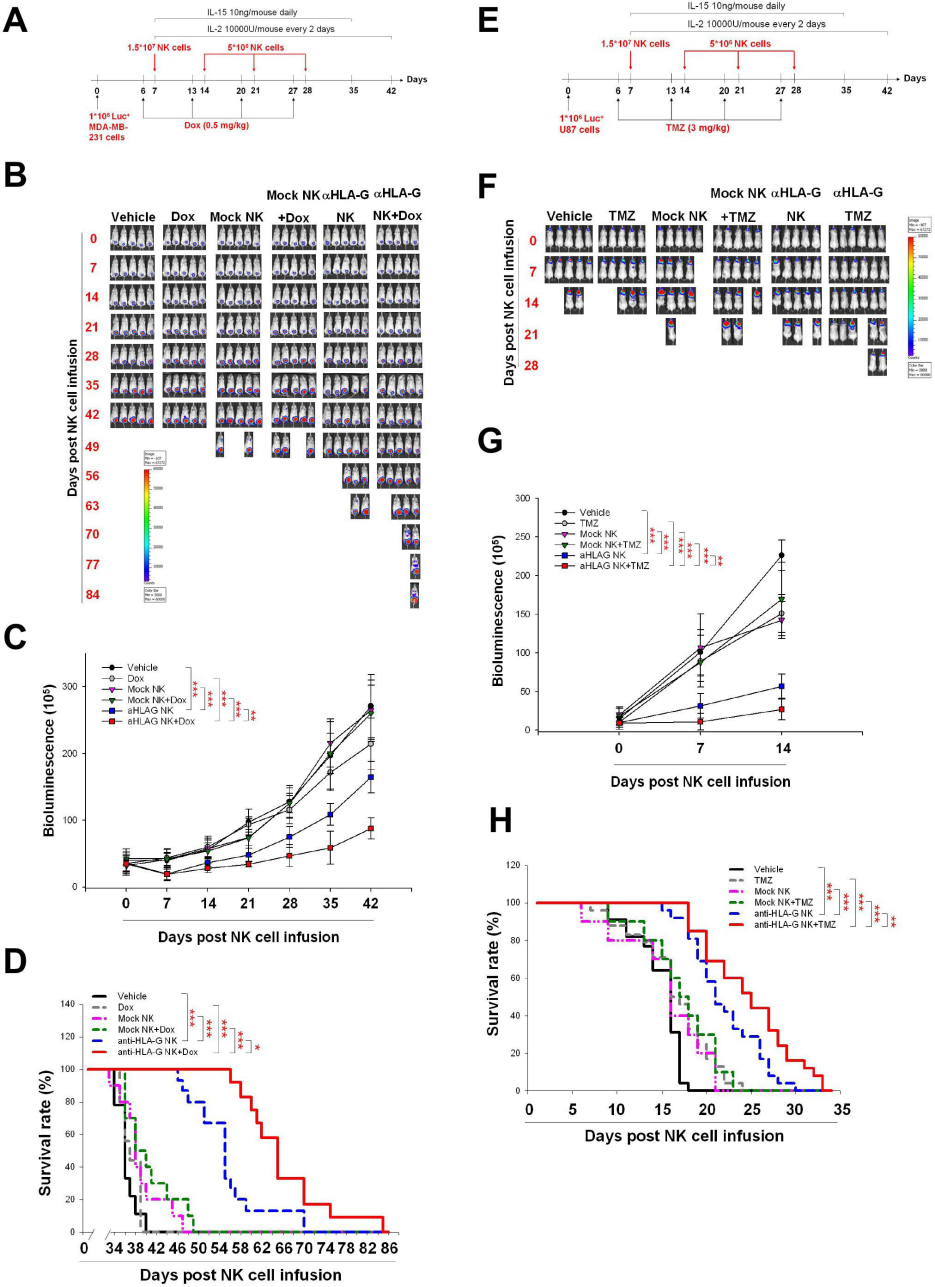
Pretreatment with Dox and TMZ enhances the in vivo antitumor activity of anti-HLA-G CAR-NK cells. (A) Treatment protocol used for the TNBC animal model. MDA-MB-231 cells (1×10^6^/mouse) were implanted orthotopically on day 0. On day 6 and for the following 3 weeks, mice received a weekly injection of normal saline or 0.5 mg/kg Dox via the tail vein. On day 7, mice were injected with normal saline or with 1.5×10^7^ mock or anti-HLA-G CAR-NK cells, followed by infusion of 5×10^6^ mock or anti-HLA-G CAR-NK cells each subsequent week for 3 weeks. (B) Representative IVIS images of MDA-MB231 tumors treated according to the protocols described in (A). Images were taken weekly. (C) Tumor progression, as measured by bioluminescence photometry. The luminoscore was calculated as the sum of flux values from the front and back views. (D) Survival was plotted using the Kaplan-Meier method. Mice were considered dead when the tumor volume exceeded 2000 mm^3^ or the greatest dimension was >1.5 cm in any direction, as measured using an electronic manual caliper. (E) Treatment protocol for the GBM animal model. U87 cells (1×10^6^/mouse) were implanted intracranially on day 0. On day 6 and for the following 3 weeks, mice were treated weekly (or not) with 3 mg/kg TMZ by oral gavage. Mice were injected with normal saline, or 1.5×10^7^ mock or anti-HLA-G CAR-NK cells, on day 7, followed by infusion of 5×10^6^ mock or anti-HLA-G CAR-NK cells each subsequent week for 3 weeks. (F) Representative weekly IVIS images of U87 tumors treated according to the protocols described in (E). (G) Tumor progression, as measured by bioluminescence photometry. (H) Survival plotted using the Kaplan-Meier method (*p<0.05, **p<0.01, ***p<0.001). CAR, chimeric antigen receptor; Dox, doxorubicin; GBM, glioblastoma; HLA-G, human leukocyte antigen G; IL, interleukin; NK, natural killer; TMZ, temozolomide; TNBC, triple-negative breast cancer.

Finally, we asked whether pretreatment with chemotherapeutic agents maximizes the antitumor activity of HLA-G CAR-NK cells in vivo. To this end, we infused HLA-G CAR-NK cells into NSG mice bearing orthotopic TNBC and GBM xenografts. Figure 7A, E illustrates the treatment protocol for these models. Infusion of HLA-G CAR-NK cells led to a significant decrease in tumor growth (figure 7B, C) and increased the survival of MDA-MB-231 tumor-bearing NSG mice (figure 7). This effect was enhanced by Dox treatment 1 day before each HLA-G CAR-NK infusion (figure 7B–D). Similar results were obtained using the orthotopic GBM model (figure 7F–H). In addition, we demonstrated that administration of an iC9 dimerisation agonist, AP20187, effectively inhibited HLA-G CAR-NK cells (online supplemental figure 1A) and disrupted their antitumor activity in vivo (online supplemental figure 1B–D). This corresponded to the decrease of CD56-positive NK cells in splenocytes from the CAR-NK-treated mice (online supplemental figure 1E). Taken together, the data showed that HLA-G-mediated redirection of CAR-NK cells exerted antitumor activity in mice bearing solid tumor xenografts, and that pretreatment with low-dose chemotherapy increases the efficacy of HLA-G CAR-NK cells.

## Discussion

This study is the first to demonstrate that targeting of HLA-G, an ICP and neoexpressed TAA, is a potential treatment for various types of solid tumors (including TNBC, GBM, PA, and OV) by CAR immunotherapy using NK cells as effectors. The anti-HLA-G construct switches the NK-inhibitory HLA-G signal to an activation signal that induces a cytotoxic killing response in NK cells engaged with target tumor cells. Furthermore, we also demonstrated a reasonable strategy for upregulating cell surface expression of HLA-G on tumor cells via pretreatment with low-dose chemotherapeutic agents; this pretreatment maximizes the antitumor efficacy of the CAR-NK cells both in vitro and in vivo. We speculate that an increase of cell surface expression of HLA-G on chemotherapy-treated cancer cells is mediated by downregulation of DMNT1 and epigenetic regulation of the *TAP-1* promoter.

To date, several preclinical studies have shown that ICPs including PD-L1,^9–12^ B7-H3,^46^ and B7-H6, are CAR targets for treatment of solid tumors.^47^ However, the most recent clinical trial of PD-L1 CAR-T (NCT03330834) cells was terminated because it caused a serious adverse event.^13^ Unlike PD-L1, which is expressed constitutively by normal epithelial cells, myeloid cells, and lymphoid cells,^48^ HLA-G is restricted to immune privileged tissues and is barely detectable in normal adult cells^49^; however, it is a neoexpressed TAA on many tumor cells.^13^ Thus, HLA-G may be an attractive target for CAR treatment of solid tumors. Here, we not only used databases and tissue arrays to confirm expression of HLA-G by tumor and normal cells, but we also used in vitro models to confirm the absence of toxicity induced by HLA-G CAR-NK cells. The data suggest that HLA-G is a safe target for CAR-based therapy. We also examined in vivo toxicity in H-2K^b^/HLA-G transgenic mice, which show tissue-specific expression of HLA-G consistent with that in humans.^50^ Again, the data suggest that CAR-NK cells targeting HLA-G are a promising anticancer therapy.

No studies have examined use of conventional chemotherapeutic agents to induce expression of specific CAR targets on tumor cells. Here, we provide the first evidence and propose possible mechanisms for chemotherapy-induced processing and transportation of HLA-G to the surface of tumor cells, which sensitize them to killing by HLA-G-specific CAR-NK cells. In addition, increased presentation of HLA-G on tumor cells results in the failure of NK cell-based immunotherapy,^20 26^ which may explain why Dox-treated HLA-G knockdown MDA-MB-231 cells were more sensitive to mock control NK-induced cytotoxic killing. This supports the concept that engineering an ‘inhibition to activation’ switch in CAR-NK cells to override immunosuppressive HLA-G signaling may be effective against multiple types of solid cancer. The anti-HLA-G scFv may compete with LILRB1 and KIR2D4 receptors on NK cells for HLA-G protein on the tumor membrane, thereby activating NK cell cytotoxicity by downregulating phosphorylated SHP-1 and upregulating phosphorylated Syk/Zap70. This competitive ability may be due to the affinity of anti-HLA-G CAR scFv for HLA-G protein on tumor cells, which is higher than that of HLA-G for inhibitory receptors on NK cells.

The use of CAR-NK cells as effector cells has several advantages over CAR-T cells, including allogeneic transfer potential, lower probability of inducing cytokine release syndrome, spontaneous tumor cytotoxicity mediated by receptors such as NKG2D, and the capacity to induce antibody-dependent cell-mediated cytotoxicity via FcγRIII.^24^ Furthermore, with respect to CAR-T cells, cytokines such as IFN-γ secreted by activated immune cells increase HLA-G expression on tumor cells, enabling them to evade antitumor immune response^51^; however, HLA-G redirected CAR-NK cells using this phenomenon to increase their antitumor efficacy.

Although the results of coculture tests did not reveal that anti-HLA-G CAR-NK exerted significant cytotoxicity, it may, however, have off-target effect on some HLA-G-expressing tissues such as splenic macrophages, thymus immune cells, and lung epitheliums when used by patients. Therefore, one of the possibilities is that HLA-G CAR-NK may damage HLA-G-expressing tissues, resulting in lymphopenia or respiratory disorder. In fact, the iC9 safety switch design was included in the HLA-G CAR construct, and it demonstrated the elimination of CAR-NK cells by the use of a CID drug, AP20187. Thus, we considered that the administration of AP20187 can be a useful solution to abort the adverse events induced by HLA-G CAR-NK. A recent study revealed that the HLA-G-expressing macrophages are of M2 phenotype and cause limited degranulation and cytotoxic capacity in NK cells through engagement of LILRB1.^22^ In addition, HLA-G-expressing immune cells such as dendritic cells and T cells have been reported to promote tumor development by inhibiting antitumor immunity.^52^ Furthermore, we found that some HLA-G-positive immune cells were present in TNBC, GBM, PA, and OV tumor sections. Thus, another possibility is that the HLA-G CAR-NK may also eliminate these immunosuppressive cells to enhance antitumor immunity in patients.

Predicting the protein levels from mRNA levels has long been fraught with unreliability and a lack of precision; hence, there are many factors that influence protein levels such as translation and degradation rates.^53^ Thus, we investigated the HLA-G protein expression patterns in various types of solid tumors and normal tissues, and then quantified these by H score. Our data showed that the expression of HLA-G protein was highly expressed in tumors only, though of limited expression in some tissues such as the spleen and thymus, except in placenta (figure 1C). This finding is consistent with that of a previous study.^17^ Therefore, IHC staining should be a better strategy to screen a target for CAR immunotherapy than mRNA-based screening. Next, 1.5×10^7^ NK cells per animal is quite a high dose when compared with that used clinically, even though it is well tolerated, as reported in a previous study.^54^ Our preliminary test showed that the single dose of CAR-NK inhibited tumor growth only in the first week after infusion, but the tumors became obviously bigger in the following week. In addition, the administration of 5×10^6^ CAR-NK cells was still an efficient dose in a TNBC PDX (patient-derived xenograft) mouse model. Furthermore, the repeated infusions of NK cells have been implemented in clinical practices.^55–57^ Therefore, we considered that the additional NK boosting is required for evaluating the efficiency of anti-HLA-G CAR-NK. It has been shown that up to 2×10^8^ cells/kg of NK cell therapies with three repeated infusions were well tolerated, even though these caused lymphodepletion.^56 57^ Thus, the translation of anti-HLA-G CAR-NK to clinical study should be tested with escalating infusing cell numbers from 1×10^7^ to 2×10^8^ cells/kg, and repeated infusions will be required.

The plasma terminal half-life of Dox is 17.3 hours^58^; that of TMZ is 2 hours^35^; that of Gem is 17 min^59^; and that of CBP is 2 hours.^60^ Lymphodepletion usually start 2–6 days before cell infusion,^61^ and the terminal half-life of cyclophosphamide and fludarabine is 16 hours^62^ and 9 hours,^63^ respectively. Thus, conditional chemotherapy for enhancing tumorous HLA-G expression should be administrated, the day before anti-HLA-G CAR-NK infusion, and lymphodepletion should be started 6 days before the infusion. This treatment protocol not only may prevent the unpredictable complications of the drugs before the subsequent CAR-NK infusion but also may enhance the tumorous HLA-G expression to strengthen the efficiency of the CAR-NK cells.

We look forward to developing HLA-G CAR-NK cells with an ‘off-the-shelf potential’ using a cryopreservation strategy. The synergistic effects of chemotherapy and HLA-G CAR-NK cells should be validated in further studies; however, we provide proof of principle that upregulating tumor cell surface expression of HLA-G by low-dose chemotherapy can increase sensitivity to CAR-NK cells and/or potentially reduce off-tumor effects in healthy tissues.

10.1136/jitc-2021-003050.supp3 Supplementary data

## Data Availability

All data relevant to the study are included in the article or uploaded as supplementary information.
