# Supplementary material for: Targeting human leukocyte antigen G with chimeric antigen receptors of natural killer cells convert immunosuppression to ablate solid tumors
Source: J Immunother Cancer. 2021 Oct 18;9(10):e003050. doi: 10.1136/jitc-2021-003050 (PMC8524382; doi:10.1136/jitc-2021-003050)
Supplement: Supplementary data [file jitc-2021-003050supp001.pdf]

Fig. S1. **DNMT1 inhibitor 5-aza regulates *TAP-1* epigenetic status and cell surface HLA-G expression** (a) MDA-MB-231, DBTRG-05MG, AsPC-1, and SKOV3 cells were treated with or without 1 μM of 5-aza (5-Aza-2′-deoxycytidine) for 48 h. The cell surface HLA-G expression levels were analysed by flow cytometry using specific antibody (b). Detection of the protein expression levels of HLA-G, TAP-1, DNMT1, β-actin, and/or E-cadherin in membrane and cytosolic fractions via immunoblotting using specific antibodies (c). Examination of epigenetic expressions of methylated or unmethylated TAP-1 gene promoter region by methylated-MSP and unmethylated-MSP PCR (d). MDA-MB-231 and SKOV3 cells were treated with or without 1 μM of 5-aza for 48 h, then co-cultured with mock or anti-HLA-G CAR-NK at an E:T ratio of 1:1 for 48 h. The cell-killing rate by NK cells was determined by flow cytometry using PI/Annexin V staining. The data are expressed as the mean ± standard error of mean of at least three independent experiments (* $p < 0.05$; ** $p < 0.01$; *** $p < 0.001$).

Fig. S2. Treatment with AP20187 eradicates anti-HLA-G CAR-transduced NK cells. (**a**) Anti-HLA-G CAR-NK cells and parental cells were treated with AP20187 (0, 1, 10, 100, 1,000, and 10,000 nM) for 24 or 48 h. Cell survival was determined by flow cytometry at the indicated times after PI staining. (**b–e**) AP20187 treatment decreased the anti-tumor activity of anti-HLA-G CAR-NK cells *in vivo*. Schematic representation of the animal study protocol. NSG

mice were orthotopically transplanted with Luc-expressing MDA-MB-231 cells (1×10^6). After 7 days, the mice were infused with anti-HLA-G CAR-NK cells (1.5×10^7) via tail vein injection and then treated (or not) with AP20187 (5 mg/kg) 4 h later (**b**). Luminescent images and signals were recorded after 7 days (**c**). Mice were euthanised and tumor samples were harvested and weighed (**d**). Mouse spleens were removed and weighed (**e**). Scale bars, 1 cm. Splenocytes were collected and the numbers of anti-HLA-G CAR-NK cells analysed by flow cytometry after staining with CD56-specific antibodies (lower right panel).
