# Supplementary figures and images for: Targeting human leukocyte antigen G with chimeric antigen receptors of natural killer cells convert immunosuppression to ablate solid tumors
Source: J Immunother Cancer. 2021 Oct 18;9(10):e003050. doi: 10.1136/jitc-2021-003050 (PMC8524382; doi:10.1136/jitc-2021-003050)

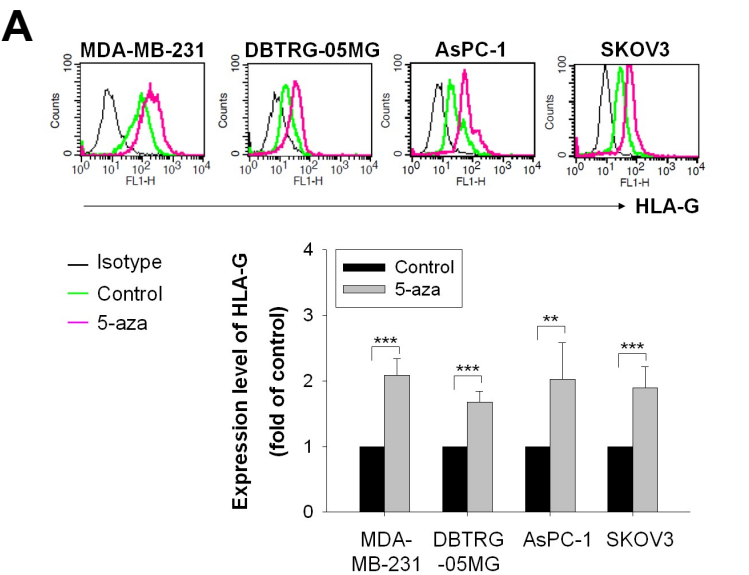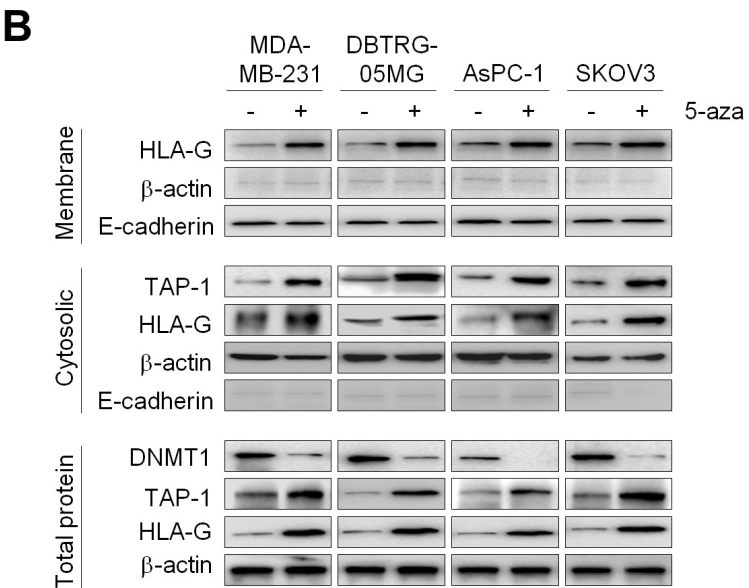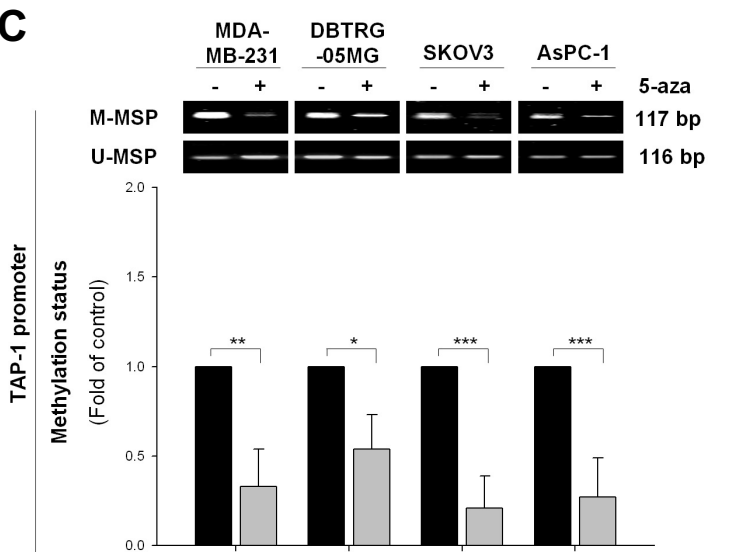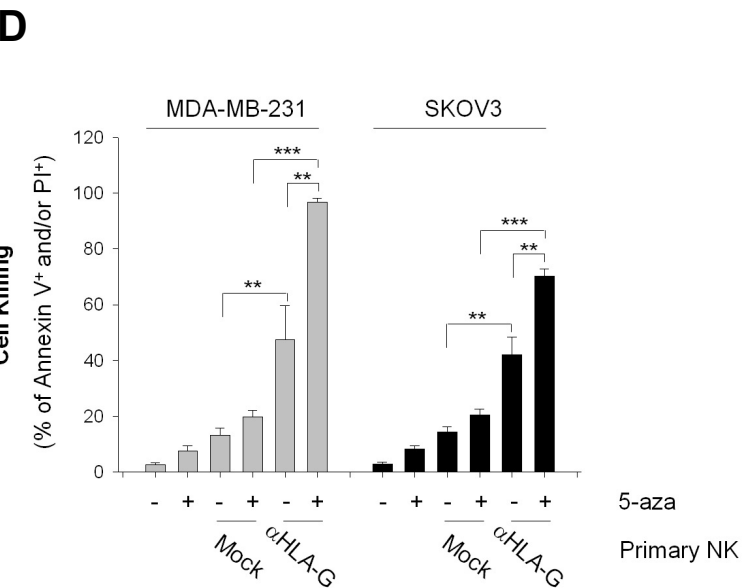

Supplement: Supplementary data [file jitc-2021-003050supp002.pdf]

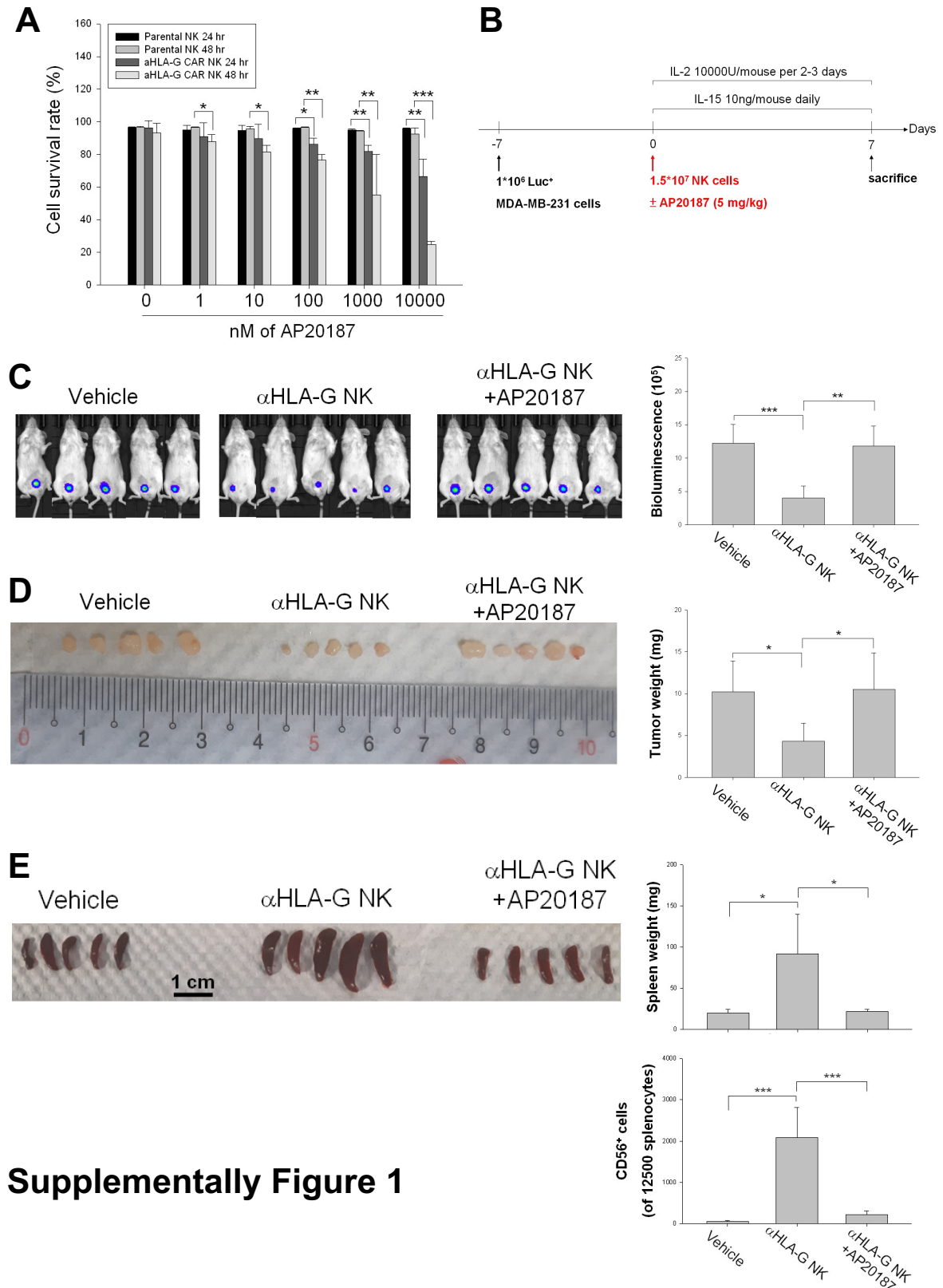

Supplementally Figure 1

Supplement: Supplementary data [file jitc-2021-003050supp003.pdf]
